# Comparison of two suture techniques on the inflammatory signs after third molars extraction–A randomized clinical trial

**DOI:** 10.1371/journal.pone.0286413

**Published:** 2023-06-23

**Authors:** Éwerton Daniel Rocha Rodrigues, Allan Vinícius Martins-de-Barros, Ariana Maria Luccas Costa Loureiro, Marianne de Vasconcelos Carvalho, Belmiro Vasconcelos

**Affiliations:** 1 School of Dentistry, Post-Graduations Program in Dentistry, University of Pernambuco (UPE), Recife, Pernambuco, Brazil; 2 Department of Oral and Maxillofacial Surgery, Hospital Universitário Oswaldo Cruz (HUOC/UPE), Recife, Pernambuco, Brazil; 3 Centro Integrado de Anatomia Patológica (CIAP), Hospital Universitário Oswaldo Cruz (HUOC/UPE), Recife, Pernambuco, Brazil; University of Catania: Universita degli Studi di Catania, ITALY

## Abstract

**Purpose:**

Wound closure technique is an operative factor that influences early post-operative complications after third molar surgery. This study investigates and compared the effectiveness of two closure techniques, primary closure and healing by second intention of the oblique incision on postsurgical discomfort after mandibular third molar surgery.

**Materials and methods:**

This is a prospective, randomized, double-blind, split mouth controlled trial. Surgical sites were divided into two groups Control group received simple sutures in both alveolar crest incision and oblique incision and intervention group received simple sutures in alveolar crest incision, while the oblique incision healed by second intention. All the patients were instructed to measure pain according to visual analogue scale (VAS) in postoperative period, swelling, mouth opening was assessed at 72h and 7 days after surgery. The wound healing was assessed on day 7.

**Results:**

Thirty-five patients, who had bilateral impacted third molars of similar surgical difficulty, were recruited. Thirty-one successfully completed the study. Patients in the second intention group had significantly less pain at 24h (p < 0.27). and 48h (< 0.001), had significantly less swelling (< 0.001) and trismus (< 0.001) and patients submitted to primary closure had a better evaluation of the Landry index (p < 0.001).

**Conclusion:**

Healing by second intention of the oblique relaxing incision by partial surgical wound closure, in our study, were superior to the primary closure in reduction of post-operative pain, swelling and trismus.

**Trial registration:**

This trial is registered at Brazilian Registry of Clinical Trials–ReBEC -UTN: RBR-5fxbqsf (https://ensaiosclinicos.gov.br/rg/RBR-5fxbqsf).

## Introduction

The extraction of mandibular third molars is the most frequent surgical intervention performed by oral and maxillofacial surgeons [1–5]. Even though a meticulous surgical technique is performed by an experienced surgeon, an inflammatory reaction is expected in the postoperative period, with symptoms such as pain, edema and trismus as result of the local inflammatory process [2, 6].

The pharmacological modulation of local and systemic mediators of pain and inflammation after removing mandibular third molars represents a challenge for oral and maxillofacial surgeons [3]; many strategies have been developed for minimizing clinical manifestations after surgery through a pharmacological approach by inhibiting the synthesis and/or release of the inflammatory mediators of acute inflammation. Among these, corticosteroids and non steroidal anti-inflammatory drug (NSAID) have shown immunosuppressive, anti-inflammatory, and analgesic effect [2–4, 7–9]. However, the use of corticosteroids or NSAIDs has been associated with some adverse effects such as gastrointestinal bleeding, renal function disturbance, a reduction in platelet function, shortness of breath, and profound hypotension [6]. So, complementary protocols, such use of phytotherapeutic drugs, photobiomodulation and wound closure techniques, have been suggested for the postsurgical therapy of third molar surgery.

There is greater drainage of the inflammatory exudate in second-intention healing, reducing edema, while this does not happen in first-intention healing [3, 7]. Some studies have demonstrated less postoperative sequelae with second intention healing [6, 7, 10–12]. However, this type of healing has the disadvantage of exposing the socket, leaving it subject to food accumulation and a prolonged healing period. Gay-Escoda [7] proposed partial closure of the flap without suturing the relieving incision after surgical extraction of lower third molars in order to reduce the disadvantages of healing by second intention.

Thus, the objective of this study is to compare whether the primary closure of the alveolus after extracting lower third molars produces a greater postoperative sequela when compared with partial suturing of the relieving incision. The null hypothesis was that there would be no difference between the two protocols analysed.

## Materials and methods

### Study design

**Fig 1:**
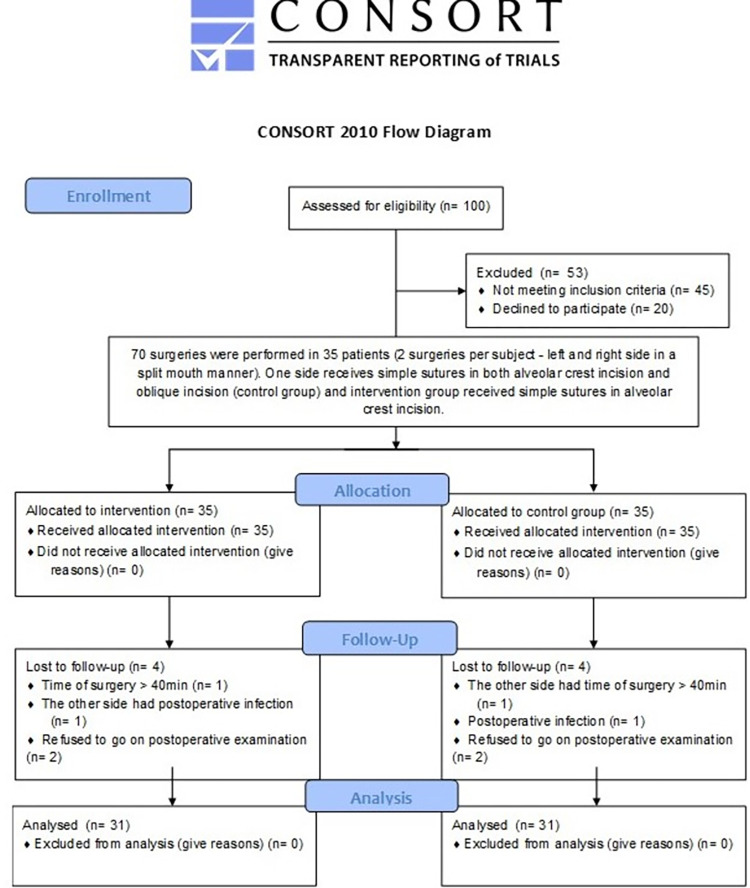
CONSORT Flowchart.

This is a double-blind randomized clinical trial study using the split mouth model conducted according to the Consolidated Standards of Reporting Trials (CONSORT). The study was approved by the Institutional Review Board of the University of Pernambuco under protocol no. 5.626.742 / CAAE 58646222.1.0000.5207 and registered in the Brazilian Registry of Clinical Trials–ReBEC (UTN: RBR-5fxbqsf). This study follows CONSORT statement (Fig 1). The individual pictured in Fig 3 has provided written informed consent (as outlined in PLOS consent form) to publish their image alongside the manuscript.

The study was Id at the Clinical Research Center of the Department of Oral and Maxillofacial Surgery of the University of Pernambuco (Recife, Brazil) in September 2022.

### Study population

The study population was composed of individuals who were indicated for surgical removal of mandibular third molars in symmetrical positions. A signed Informed Consent Form was obtained from each enrolled participant after informing them of the research purposes, risks and benefits.

The inclusion criteria were set as: 1) individuals of both genders, 2) aged between 18 and 50 years, 3) classified as ASA I according to the American Society of Anesthesiologists, 4) who have both mandibular third molars in similar positions according to the Pell and Gregory and Winter classifications, 4) absence of pericoronitis or signs of inflammation. The exclusion criteria were: 1) participants who presented a disease or systemic condition that contraindicated the procedure; 2) who were in gestation or lactation period; 3) who used chronic medications or other substances that influence the inflammatory response or bone metabolism; 4) or who refused to participate in the data collection steps were excluded from the study.

### Surgical procedure and set of interventions

According to the split mouth model, each participant underwent two surgical procedures, one for each side, with a wash-out interval of at least two weeks between them. The surgical procedures were standardized and performed by an expert Oral and Maxillofacial Surgeon. The participants were given 8mg of dexamethasone orally one hour before the procedures. Local anesthesia of the inferior alveolar, lingual and buccal nerves was performed using an anesthetic solution of lidocaine 2% with epinephrine 1:100.000 (Alphacaine®—DFL). The impacted third molar was extracted with elevators and the incision was made according to the randomization. When necessary, osteotomies and odontosection were performed with hand-pieces and carbide burs No. 702 (Dentsply International, New York, USA) under sterile saline solution irrigation.

**Fig 2:**
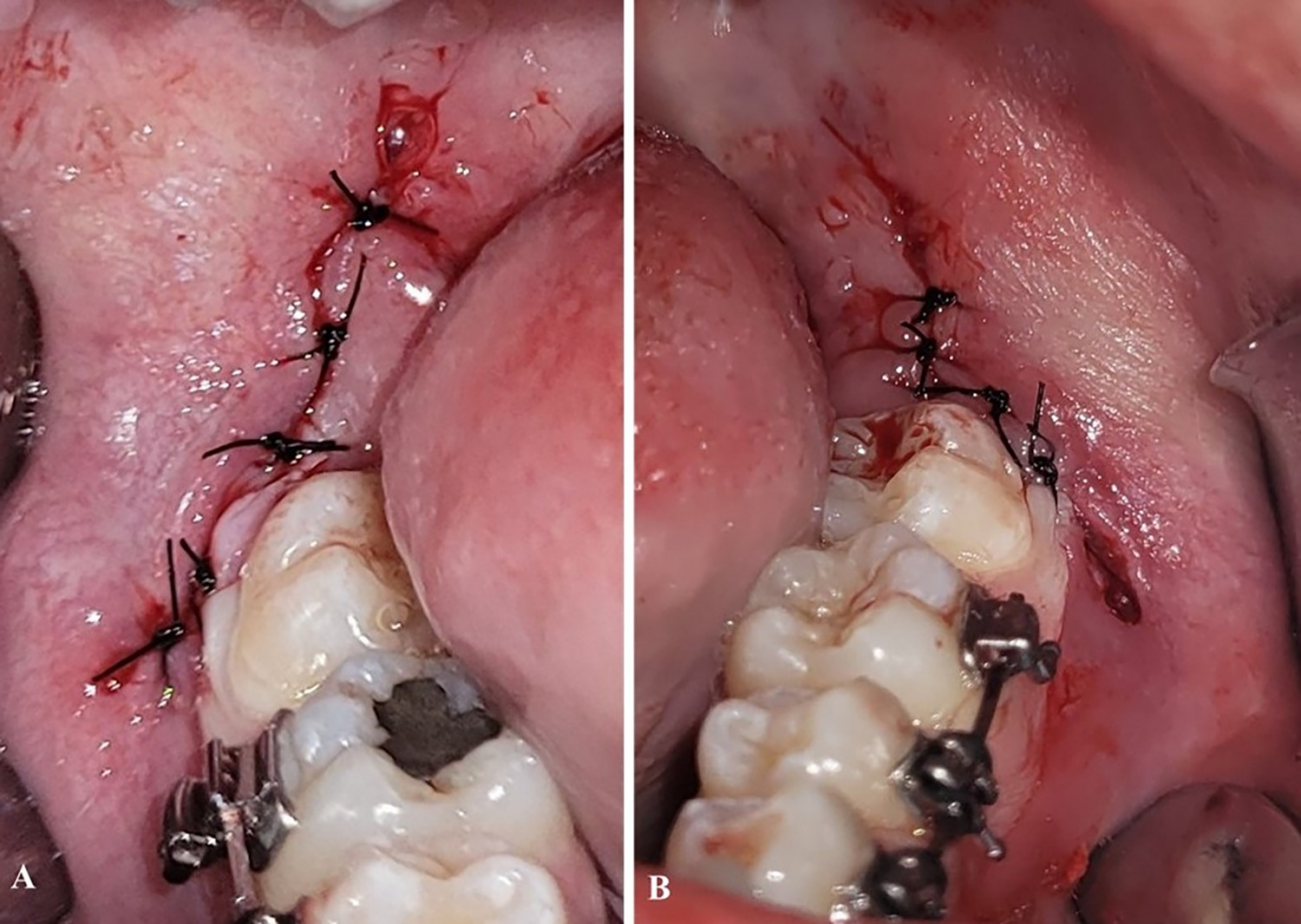
**A**—simple sutures in both alveolar crest incision and oblique incision. **B—**simple sutures in alveolar crest incision, while the oblique incision was healed by second intention.

The primary predictor variable was the surgical wound closure technique. The participants were randomized and allocated to receive either primary closure (control group) or partial closure (intervention group) of the surgical wound, both performed with 3–0 silk threads. The control group received simple sutures in both alveolar crest incision and oblique incision (Fig 2A). The intervention group received simple sutures in alveolar crest incision, while the oblique incision was healed by second intention, without sutures (Fig 2B). After the surgical procedure, each participant received postoperative instructions and 10 tablets of 750mg acetaminophen (EMS Sigma Pharma Ltda., Hortolândia/SP, Brazil) and were instructed to use them as an oral analgesic if there was any need for pain relief.

### Randomization

An external researcher performed the randomization. After the eligibility assessment and prior to the first procedure, randomization of the intervention (primary closure or partial closure of the surgical wound) and of the first side to be operated (right or left) were performed with the assistance of the simple randomization service from Sealed Envelope™ (available on: https://www.sealedenvelope.com/). The second surgical procedure was performed on the contralateral side of the first procedure after the wash-out period, with a different surgical wound closure technique than the used in the first procedure.

### Blinding

The participants and the examiner were not aware of the surgical wound closure technique adopted in each intervention, thus providing double blinding to the study.

### Evaluation of outcome variables

The main outcome variables were postoperative pain, swelling, trismus and level of surgical wound healing. All outcome variables were clinically and prospectively evaluated during the study by a blinded and previously trained examiner.

Postoperative pain was measured using the Visual Analogue Scale (VAS) ranging from 0 (absence of pain) to 10 (severe pain). Subjects were instructed to measure pain according to VAS at 30 minutes, 2h, 4h, 6h, 8h, 12h, 16h, 24h, 48h and 72h of postoperative period. Additionally, the total number of analgesic tablets consumed up to the first 72h postoperative was also recorded.

**Fig 3:**
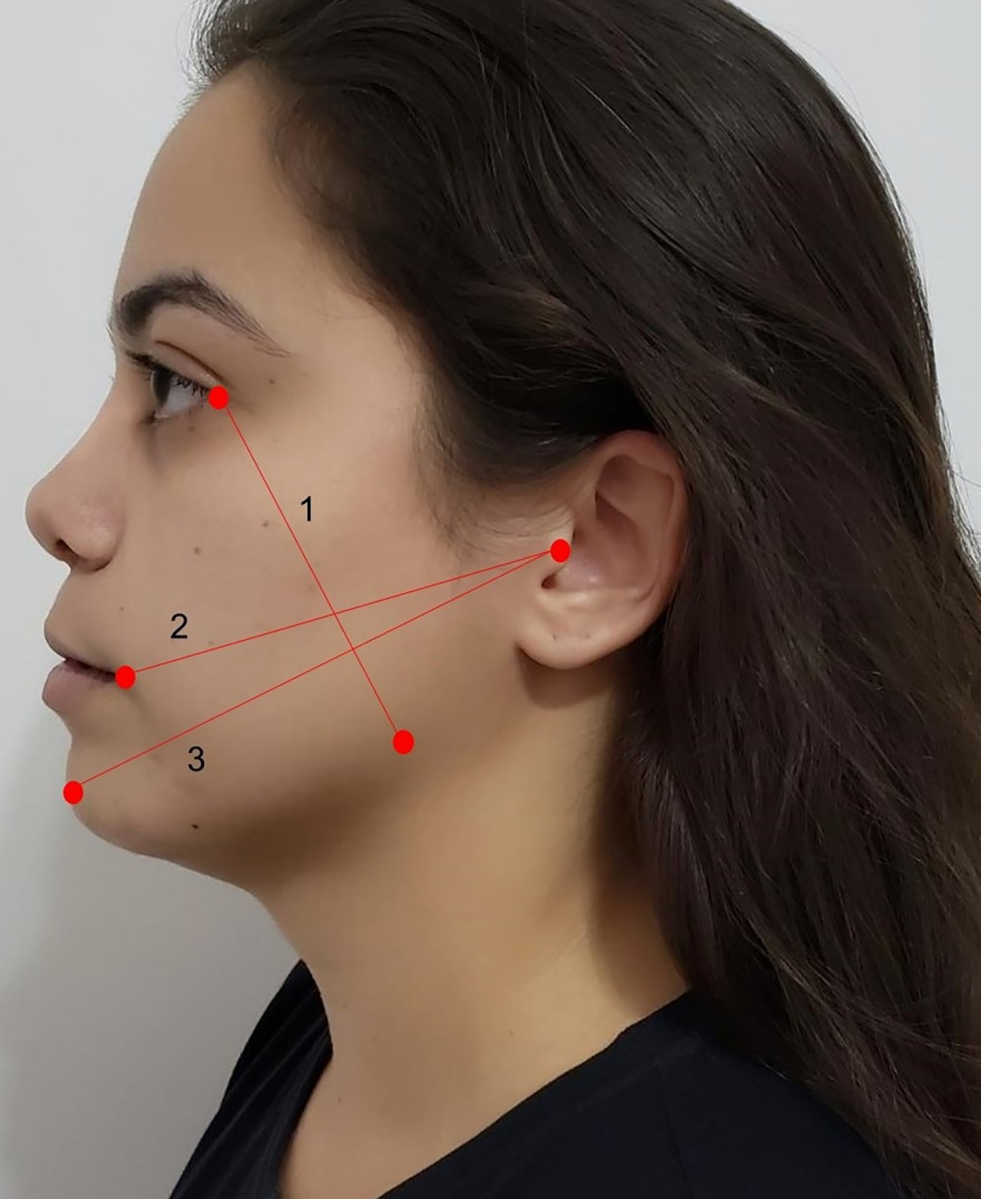
Facial measurements used to assess edema.

Swelling and trismus and the measurements were performed prior to the surgical procedure (baseline) and at 72h and 7 days after surgery. Swelling was evaluated by measuring the distance between reference points on the participant’s face with a tape measure. Three facial measures were obtained from each patient: 1) The distance from angle of the mandible to the external cantus of the eye; 2) The distance from the tragus to labial commissure; and 3) The distance from the tragus to the chin. The sum of the three measures was computed and the swelling was considered the variation between the preoperative baseline and the postoperative measures. The illustration of reference points is shown in Fig 3.

Trismus was evaluated based on the mouth opening limitation. The interincisal distance during maximum mouth opening was measured in millimeters with a digital caliper taking the incisal edge of the upper and lower left central incisors as reference. The mouth opening limitation was determined by the difference in millimeters between the preoperative baseline and postoperative interincisal distance measures.

Surgical wound healing was evaluated on the 7th postoperative day by the Landry index [13]. The Landry index rates the surgical wound healing process as “very poor”, “poor”; “good”; “very good”; or “excellent” according to tissue color, bleeding on palpation, presence of granulation tissue, incision margins and suppuration.

### Sample size

The sample size was calculated in the OpenEpi 3.01 program through the mean differences of the outcome variable “swelling” between the study groups in a pilot study (mean difference set as 2.61, with standard deviation of 2.57 and 2.31 for groups primary and secondary closure, respectively), establishing a 95% confidence interval and 80% power, with type I error of 0.05. On the basis of these values, a minimum sample size of 28 patients was calculated to be necessary, with this number being greater than the sample sizes calculated for the other variables (pain and trismus).

### Statistical methods

The database was built in the IBM SPSS® software version 20.0 platform (IBM Corp., Armonk, NY, USA). Descriptive and inferential statistical analyses were performed. The descriptive data analysis was presented as absolute and relative frequencies and means. The Kolmogorov-Smirnov test was used to test the normality of the quantitative variables for the inferential statistics. The t-test for paired samples was used to compare the means of quantitative variables (swelling and trismus), while the Wilcoxon Signed Rank test was used to assess differences in ordinal variables (pain and Landry index) between the intervention groups (primary vs. partial suturing of the relieving incision). A significance level of 5% (p < 0.05) was adopted for all statistical tests.

## Results

Tirty-five patients underwent surgery and four were excluded from the analysis. One of the patients was excluded due time of surgery was moren than forty minutes, one due had postoperative infection e and two refused to gon on postoperative examination. Demographic and clinical data of the participants (gender, age, skin color) and the degree of impaction and the position of the mandibular third molars according to Pell and Gregory and Winter classifications are described in Table 1.

**Table 1:** Demographic and clinical data of the participants of the clinical trial.

Variável	Group	
	Primary closure	Partial closure
**Sex**		
Men	12	
Women	19	
**Age (mean ± SD)**	22.04 ± 2.45	
**Surgery time (mean ± SD)**	21.4 ± 1.6	21.6 ± 2.1
**Pell & Gregory classification**	**n (%)**	**n (%)**
I A	5 (16.1)	5 (16.1)
II A	8 (25.8)	8 (25.8)
I B	7 (22.5)	7 (22.5)
II B	6 (19.3)	6 (19.3)
I C	5 (16.1)	5 (16.1)
**Winter classification**		
Mesioangular	8 (25.8)	8 (25.8)
Horizontal	5 (16.1)	5 (16.1)
Vertical	18 (58.06)	18 (58.06)

SD, standard deviation.

Participants submitted to partial closure of the surgical wound consistently showed lower scores of pain throughout postoperative evaluation. Statistically significant differences (p ≤ 0.05) were observed in 2h, 8h, 24h, 48h and 7 days, as shown in Table 2.

**Table 2:** Comparison of pain scores between groups in different moments of postoperative period.

Pain Level (VAS)			
Postoperative time	Primary closure (mean ± SD)	Partial closure (mean ± SD)	*p* Value (Wilcoxon test)
30 min.	0.00 ± 0.00	0.00 ± 0.00	1.000
2h	0.13 ± 0.34	0.00 ± 0.00	**0.0046** *
4h	0.81 ± 1.13	0.52 ± 0.81	0.359
6h	1.90 ± 1.51	1.42 ± 1.19	0.097
8h	1.61 ± 1.66	0.74 ± 1.09	**0.025** *
12h	1.61 ± 1.56	1.39 ± 1.17	0.478
16h	1.06 ± 1.06	0.94 ± 0.77	0.532
24h	2.19 ± 1.55	1.29 ± 1.21	**0.031** *
48h	2.61 ± 1.47	1.42 ± 0.99	**< 0.001** *
72h	1.52 ± 1.33	1.42 ± 0.99	0.926
7 days	0.26 ± 0.44	0.03 ± 0.18	**< 0.001** *

VAS, Visual Analogue Scale; SD, standard deviation

* *p* < 0.05.

Table 3 shows swelling, trismus and surgical wound healing measures during the postoperative period. Swelling and trismus means at 72h postoperative were significantly higher in the primary closure group when compared to the partial closure group (p<0.001).

**Table 3:** Comparison of swelling and trismus and surgical wound healing (Landry index) between groups in different moments of postoperative period.

Postoperative time	Primary closure (mean ± SD)	Partial closure (mean ± SD)	*p* Value
**Swelling**			Paired t-test
72h	9.29 ± 2.57	6.68 ± 1.95	**< 0.001** *
**Trismus**			Paired t-test
72h	8.90 ± 2.24	7.29 ± 2.31	**< 0.001** *
**Landry Index**			Wilcoxon test
7 days	4.29 ± 0.58	2.84 ± 0.68	**< 0.001** *

SD, standard deviation

* *p* < 0.05.

Based on the Landry index on the 7th postoperative day, the surgical wound healing in the primary closure group varied from good to excellent, with most participants presenting with very good (58.1%) or excellent (35.5%) healing. In contrast, most of the participants in the partial closure group presented poor (32.3%) or good (51.6%) healing (Table 4). The differences were statistically significant (p<0.001).

**Table 4:** Landry index at 7 days postoperative in both primary and partial closure groups.

Variables	Primary closure		Partial closure	
Landry index	n	%	n	%
1 –very poor	00	0.0	00	0.0
2 –poor	00	0.0	10	32.3
3 –good	02	6.5	16	51.6
4 –very good	18	58.1	05	16.1
5 –excelente	11	35.5	00	0.0

## Discussion

The aim of this study was to evaluate the course of signs and symptoms of infla–mation—pain, swelling and trismus, in addition to soft tissue healing in two types of closure techniques: primary, secondary after lower third molar surgery. Flap design appears to be not associated with periodontal complications [8]. However, some studies have shown the benefits of using a drain [11, 12, 14, 15], partial closure of the suture[9, 15–19] and sutureless [20, 21]. These authors proved that there are fewer inflammatory signs (pain, edema and trismus) in patients in which the alveolus is partially closed or which allows a drainage route. Healing by second intention of the oblique relaxing incision, in our study, were superior to the primary closure in reduction of post-operative pain, swelling and trismus.

The reduction of postoperative discomfort after removal of third molars represents a challenge. Anti-inflammatory agents have been used preemptively to reduce the inflammatory signs and symptoms resulting from this surgical procedure [2]. However the use of corticosteroids or NSAIDs has been associated with some adverse effects such gastrointestinal bleeding, renal function disturbance, a reduction in platelet function, shortness of breath, and profound hypotension [5, 6]. Alternative protocols have been suggested with the aim of reducing postoperative discomfort following third molar surgery without causing adverse effects. Phytotherapeutic drugs, laser therapy, piezosurgery, knesiotherapy have been demonstrated positive effects on modulation of localized edema, and pain following extraction of mandibular impacted third molars [1, 2]. Our research has focused on management of soft tissue healing because it is a simromisingomissing results.

We used a visual analogue scale (VAS) to assess pain severity. Thus, the side where the secondary closure was performed had the lowest pain scores, with a statistically significant difference in the 2, 8, 24, 48-hour evaluations and in the 7-day evaluation. These findings are in agreement with the findings of Pasqualini et al. [10], Holland and Hindle [11] and Dubois et al. [17]. In addition, according to Balamurugan and Zachariah [3], post-operative care and hygiene of a secondary closure site were more easily performed by the patient. A possible explanation for this finding would be lower inflammatory exudate retention. Exudate retention is less in partial closure because there is more space for the release of inflammatory exudate compared to the multiple suture technique.

A systematic review published by Azab et al. [22] suggest a significant reduction in pain scores on the first postoperative day, favoring secondary closure. Then, an insignificant reduction on the third and seventh postoperative day favoring secondary closure. However, a high risk of bias was found, mainly for postoperative assessments on days 1 and 3.

In this clinical trial, measurements of the edema level were performed in the preoperative period, in the immediate postoperative period, after 72 hours and on the seventh day after tooth extraction. Preoperative and immediate postoperative measurements were similar. This is due to the split-mouth design, in which each patient is their own control, and because only individuals with third molars in similar positions were included, aiming at similar surgical trauma between the experimental and control sides. In line with the findings by Pasqualini et al. [10], the edema peak occurred 72 hours after the surgery, with the group and the partial suture showing the smallest measurements (p<0.001). Maria et al. [16] and Singh et al. [23] observed the edema peak occurring 24h after surgery, and Osunde, Saheeb and Adebola [15] on the second postoperative day. The differences may have resulted from variations in the individual inflammatory response.

A systematic review by Carrasco-Labra et al. [24] failed to show statistically significant differences in mean percentage of trismus between groups. Pooled analysis showed that the mean difference in trismus on the third postoperative day was 3.72 in favor of closure, and the mean difference on the seventh day was 2.35 mm in favor of partial closure. In this clinical trial, the mean decrease in mouth opening was 8.90 for the primary closure and 7.29 for the secondary closure (p<0.001). These findings are consistent with findings found in the literature [10, 15, 17].

Several methods have been suggested for performing partial closure after tooth extraction, from not performing the suture [20, 21], to using drains [11, 14, 25], partial suturing [4, 15] and excision of the coronal portion of the mucosa [1, 3, 10, 15]. The sutureless technique is a procedure limited to small incisions excision of the mucosa [10, 26]. Drain insertion and excision of the coronary portion of the mucosa increase trauma and surgical time, in addition to the drain being a type of foreign body [24]. Furthermore, leaving the coronary portion without sutures can lead to an accumulation of food remains and the possibility of socket infection. Healing by second intention of the relaxing incision, as proposed by some authors [3, 7, 26], would be an option to overcome this problem.

This study showed a significant reduction in pain, edema and trismus in the group in which the relaxing incision was not sutured compared to hermetically closed sockets, and these findings agree with the findings of Balamurugan and Zachariah [3]. Gay-Escoda et al. [7] published similar findings, with the group that did not have the relaxing incision sutured showing lower scores for pain, edema and trismus, although without a significant difference in relation to the control group. Soft tissue healing was evaluated using the Landry index [13]. Moreover, better soft tissue healing was observed at one week after surgery in sockets that were hermetically sealed compared to the experimental group. Another possible disadvantage of this suture modality would be postoperative bleeding [18]. However, some studies have not shown a higher rate of postoperative bleeding in alveoli that were partially closed [4, 10, 20].

A split-mouth design was selected because the patients act as their own controls. Which increases the power of the study because of the removal of interpatient variability [27]. Although the need to recruit patients with third molars in a symmetrical position made it difficult for us to recruit. The assessment of postoperative pain is the major limitation of this study. The level of pain was recorded on a self-reported form, which may lead to some inaccuracy and inter-individual variation in data recording. The results of this preliminary study are promissing, however our findings are only from a randomized clinical trial of Brazilian subjects. So further research is required to provide a better understanding of the potential benefits of partial closure in postoperative therapy following impacted third molar surgery.

## Conclusion

Healing by second intention of the oblique relaxing incision by partial surgical wound closure combines the advantages of primary closure, in which the coronal portion of the socket is sealed, and secondary closure, which allows an active drainage pathway for inflammatory exudate. Therefore, although a slight delay in the surgical wound healing is expected when the partial closure technique is used, the postoperative pain, swelling and trismus levels are significantly decreased when compared to primary closure.

## Supporting information

S1 Checklist CONSORT checklist. (DOC)
